# Mental Health Treatment Involvement and Religious Coping among African American, Hispanic, and White Veterans of the Wars of Iraq and Afghanistan

**DOI:** 10.1155/2011/192186

**Published:** 2011-07-18

**Authors:** David S. Greenawalt, Jack Y. Tsan, Nathan A. Kimbrel, Eric C. Meyer, Marc I. Kruse, David F. Tharp, Suzy Bird Gulliver, Sandra B. Morissette

**Affiliations:** ^1^VISN 17 Center of Excellence for Research on Returning War Veterans, Department of Veterans Affairs, Waco, TX 76711, USA; ^2^Texas A&M Health Science Center, College of Medicine, Bryan, TX 77807, USA

## Abstract

Although racial/ethnic differences have been found in the use of mental health services for depression in the general population, research among Veterans has produced mixed results. This study examined racial/ethnic differences in the use of mental health services among 148 Operation Enduring/Iraqi Freedom (OEF/OIF) Veterans with high levels of depression and posttraumatic stress disorder (PTSD) symptoms and evaluated whether religious coping affected service use. No differences between African American, Hispanic, and Non-Hispanic white Veterans were found in use of secular mental health services or religious counseling. Women Veterans were more likely than men to seek secular treatment. After controlling for PTSD symptoms, depression symptom level was a significant predictor of psychotherapy attendance but not medication treatment. African American Veterans reported higher levels of religious coping than whites. Religious coping was associated with participation in religious counseling, but not secular mental health services.

## 1. Introduction

Research indicates that African Americans and Hispanics underutilize mental health services compared with Non-Hispanic whites. According to the National Comorbidity Survey (NCS) and the National Comorbidity Survey-Replication (NCS-R), among those with a mental disorder, African Americans are half as likely as whites to receive psychiatric treatment when controlling for the severity of the disorder [1]. African Americans are also less likely than other racial/ethnic groups to receive treatment for a mental disorder through specialty mental health or general medical services [2]. A recent review indicated that Hispanics, as compared to Non-Hispanic whites, also underutilize mental health services and are less likely to receive adequate mental health treatment [3]. 

Racial and ethnic differences in use of mental health services have also been shown for specific disorders, such as depression. African Americans are less likely than whites to access mental health treatment for depression, and of those who receive treatment, African Americans are less likely than whites to receive adequate care [4, 5]. A recent national study found that Hispanics, compared to Non-Hispanic whites, were also less likely to receive mental health treatment for depression during the period from 2000–2002 [6]. 

Whereas data from general community samples have revealed differences in mental health service utilization between minorities and whites [1, 2, 6–8], research with US military Veterans has produced mixed findings regarding utilization rates, access to adequate care, and treatment adherence. A study that examined all mental health treatment (VA and outside treatment) found that minority Veterans underutilized outpatient mental health services relative to whites [9]. Rosenheck and Fontana [10] found that African American and Mexican-American Veterans were less likely than whites to use mental health services outside of the VA. A study assessing outpatient care for depression at 14 VHA hospitals in the Northeast found that African-Americans, as compared with other Veterans, were less likely to receive adequate pharmacotherapy for depression [11]. Further, African American Veterans who received pharmacotherapy for bipolar disorder through the VA reported lower medication adherence than did whites [12]. In contrast to studies finding lower mental health service use among African Americans, a large study (*N* = 41,412) of Veterans newly diagnosed with a depressive disorder found that African Americans, as compared to whites, were more likely to receive guideline-compliant levels of psychotherapy through the VA [13]. Other studies have found no differences in the use of VA mental health services between minorities and whites [10, 14–17]. 

Of particular relevance are utilization rates of today's Veterans, who are undergoing multiple deployments and have high rates of mental disorders [18, 19]. Two recent studies examining mental health utilization rates among Operation Enduring Freedom and Operation Iraqi Freedom (OEF/OIF) Veterans did not find racial/ethnicity differences [16, 17]. However, these studies did not control for levels of depression and posttraumatic stress disorder (PTSD) symptoms, an indirect index of need for services. Analyses that include indicators of need have frequently demonstrated differences in utilization based on race or ethnicity [1, 5, 9]. In addition, Seal et al. [17] focused primarily on treatment for PTSD rather than for depression or other mental disorders. Given the high rates of both PTSD and depression among OEF/OIF Veterans [20, 21], mental health treatment research should consider both disorders.

Moreover, research examining the use of mental health services often does not assess the use of counseling through a pastor or other religious figure, which is notable, given that 30 percent of Americans surveyed about counseling preferences said they would rather use religious than nonreligious counseling [22]. In particular, people who report high levels of religious coping or religiosity (e.g., frequent prayer) may be more likely to seek religious counseling and less likely to seek secular mental health treatment when dealing with psychological difficulties. For example, people who attend church more often are more likely to prefer therapeutic help from ministers or church staff rather than specialized mental health providers [22, 23].

It follows that a large proportion of Veterans may prefer religious counseling over specialty mental health services at the VA. Nearly 30 percent of Operation Enduring Freedom/Operation Iraqi Freedom (OEF/OIF) Veterans believe that religious counseling would be more effective than conventional mental health counseling for their problems [24]. Because religious counselors may rarely refer their counselees to specialized mental health resources [25], the use of religious counseling may reduce the use of secular mental health services. 

Race and/or ethnicity may be related to the use of religious counseling. Compared to whites, African Americans are more likely to find spiritual counseling acceptable and less likely to find prescription medication acceptable for the treatment of depression [26]. Traditionally, African Americans have had limited access to formal mental health services compared to whites and may have preferred religious counseling, in part, because of reduced expense [27]. African American churches offer more mental health services than white churches [28]. A recent qualitative study suggests that African American culture has stressed self-reliance and religious coping as an alternative to specialized mental health treatment [29]. Similarly, Hispanic Americans often prefer to speak with a priest or member of the clergy rather than a psychologist or psychiatrist when they experience psychological problems, especially if they are acculturated toward a traditional Hispanic culture rather than American culture [30, 31]. Hispanic Veterans who were not currently using mental health services at the VA preferred to seek help from a priest or friend rather than conventional mental health providers when they experienced emotional distress [32]. 

Racial or ethnic differences have also been observed in religious coping, such as the use of prayer and finding comfort in one's religion. Compared to whites, African Americans consistently report higher use of positive religious coping (e.g., prayer, having faith in God) [33–37]. In addition, there is some evidence of greater religious coping among Hispanics as compared to non-Hispanic whites [36, 38, 39].

A number of studies indicate that religious coping and religiosity are associated with increased well-being, psychological recovery, and lower depression symptoms, regardless of race [33, 40–43]. In addition, reduced levels of depression and general psychopathology have been found among African Americans [44, 45] and war Veterans [46] who report greater use of religious coping or religious guidance in their daily life.

The current study expands upon the existing literature by examining racial/ethnicity differences in mental health service utilization among OEF/OIF Veterans after controlling for depression and PTSD symptom levels. The study also contributes to existing literature by examining racial/ethnic differences in the use of secular versus religious counseling and the role of religious coping on use of mental health services among OEF/OIF Veterans. Whereas one study of Vietnam Veterans [10] assessed racial/ethnic differences in use of religious counseling, no published studies of OEF/OIF Veterans have examined religious coping or use of religious counseling. Given a lack of previous research on religious variables among Veterans and mixed findings regarding racial/ethnic disparities in mental health service use, the following exploratory hypotheses were tested through secondary data analysis.

**Hypothesis 1**

Whites, as compared to African Americans or Hispanics, would be more likely to attend specialty mental health treatment.

**Hypothesis 2**

African Americans and Hispanics, compared to whites, would be more likely to seek religious counseling.

**Hypothesis 3**

Compared to whites, African American and Hispanic Veterans would report higher levels of religious coping.

**Hypothesis 4**

Higher levels of religious coping would be associated with increased likelihood of seeking religious counseling.

**Hypothesis 5**

Higher levels of religious coping would be associated with lower levels of specialty mental health treatment.

## 2. Methods

### 2.1. Participants

Secondary data analyses were conducted using preliminary data from OEF/OIF Veterans taking part in Project SERVE (Study Evaluating Returning Veterans' Experiences; PI: Morissette), an ongoing, longitudinal study of returning OEF/OIF Veterans. Veterans were recruited from the three main campuses of the Central Texas Veterans Health Care System (CTVHCS)—two medical centers and one large outpatient clinic. Eligible participants included OEF/OIF Veterans age 18 years or older who spoke English, were able to comprehend and complete the assessment battery and were willing to complete required follow-up assessments. Additionally, at the time of the baseline assessment, Veterans taking or discontinuing psychotropic medications needed to demonstrate medication stability, as indicated by (1) taking a prescribed selective serotonin reuptake inhibitor or monoamine oxidase inhibitor for more than three months; (2) taking a prescribed anxiolytic or beta-blocker for more than one month; or (3) more than one month medication discontinuation or “wash-out” for all medications. In keeping with medication stabilization, patients in psychotherapy were required to demonstrate stability as indicated by greater than three months in psychotherapy or at least one-month therapy “wash-out” (i.e., one month or more since last therapy session). These latter two criteria were instated to ensure that symptoms assessed during the baseline assessment were due to an underlying psychiatric condition and not due to the temporary effects of starting or stopping medications and/or psychotherapy. Participants were excluded if they had plans to relocate from the Central Texas catchment area within four months of starting the study; met criteria for a diagnosis of schizophrenia, another psychotic disorder, or bipolar disorder; reported current hallucinations or delusions; or reported current suicidal or homicidal risk that warranted crisis intervention. Participants were recruited through direct mailings, through advertising at enrollment sites and Veterans' service organizations, and through referral from VA primary care staff, mental health staff, and OEF/OIF coordinators. Recruitment efforts were directed toward obtaining a sample comprised of approximately 75% of participants with a current or lifetime psychiatric diagnosis and 25% with no diagnoses. 

Data were available for 188 participants who had completed the baseline assessment for Project SERVE to date. Only participants who reported their racial/ethnic background as African American, Hispanic, or white (non-Hispanic) were retained for analyses in the current study. Participants who reported that they were Asian, Hawaiian/Pacific Islander, Native American, biracial (with the exception of Hispanic and white), or other were excluded due to inadequate subsample size (*n* = 17). Also excluded from analyses were 3 participants who did not provide racial/ethnic information, 4 participants who did not provide information on the primary outcome measure (use of mental health services), 8 participants who did not complete either the Beck Depression Inventory-II (BDI-II [47]) or the Posttraumatic Stress Disorder Symptom Checklist-Military (PCL-M [48]), and 8 participants who had missing sociodemographic data (e.g., annual income). Participants who identified as both Hispanic and white (*n* = 19) were categorized as Hispanic. The final sample consisted of 148 OEF/OIF Veterans.

The composition of the final sample was as follows: 125 males (84.5%) and 23 females (15.5%); 78 Non-Hispanic whites (52.7%), 30 Non-Hispanic African Americans (20.3%), and 40 Hispanics (27.0%). The median annual income range for the sample was $30,000–49,999. See Table 1 for sample characteristics.

### 2.2. Procedure

As part of the larger Project SERVE longitudinal study, participants were screened by telephone to determine initial eligibility and scheduled for a comprehensive baseline assessment. At the beginning of the baseline assessment, informed written consent was obtained. The baseline assessment lasted approximately five hours, after which final eligibility was confirmed. Participants were compensated for their time ($60–75 plus $5 for lunch).

### 2.3. Measures

#### 2.3.1. Demographics

Participants completed a demographics questionnaire designed specifically for the study that assessed race/ethnicity, age, military service, years of education, hours worked per week in the past 3 months, and annual income in the last 12 months.

#### 2.3.2. Treatment Involvement

A Treatment Involvement Form (TIF), a self-report measure created specifically for the study, assessed each participants' number of individual psychotherapy, group psychotherapy, religious counseling, and mental health medication management appointments during the past 4 months.

#### 2.3.3. Beck Depression Inventory-II (BDI-II [47])

The BDI-II is a 21-item self-report measure that assesses the severity of depressive symptomatology. Items are rated on a scale from 0 to 3, with higher scores indicating greater symptom severity. The BDI-II was designed to be more compatible with the DSM-IV depression symptomatology and temporal criteria than the original BDI. The BDI-II has adequate criterion validity [49] and has demonstrated high levels of internal consistency (Cronbach's alpha = .91) [50]. The BDI-II is an effective depression screening instrument among diverse racial and ethnic groups [51]. Internal consistency for the current study sample was Cronbach's alpha = .94.

#### 2.3.4. Posttraumatic Stress Disorder Symptom Checklist-Military Version (PCL-M [48])

The PCL-M is a 17-item self-report measure that assesses PTSD symptoms during the previous month among military servicemen and women. Subjects rate each item on a 5-point Likert scale ranging from 1 (“not at all”) to 5 (“extremely”), with higher score indicating greater symptom severity. Prior research has demonstrated test-retest reliability of  .96 and  .88, with test-retest intervals of 2-3 days and 1 week, respectively [52, 53]. The PCL is positively correlated with the Clinician Administered PTSD Scale for DSM-IV (CAPS) at  .92 [52]. Using a cutoff score of 50 produces good sensitivity (.78) and specificity (.82) for a diagnosis of PTSD in the past month [54]. Internal consistency for the current sample was  .97.

#### 2.3.5. Brief COPE [55]

The Brief COPE assessed coping strategies participants used during the most recent four months when confronting stressful live events. The Brief Cope is a 28-item, shortened version of the original 60-item self-report COPE measure. The Brief COPE consists of 14 subscales, each comprised of two items. Carver [55] reported the following subscale alphas: active coping (.68), planning (.73), positive reframing (.64), acceptance (.57), humor (.73), religion (.82), using emotional support (.71), using instrumental support (.64), self-distraction (.71), denial (.54), venting (.50), substance use (.90), behavioral disengagement (.65), and self-blame (.69). Factor structures generated from the Brief COPE were similar to that of the full COPE. The full COPE has demonstrated good convergent and discriminant validity [56]. Internal consistency for the religious coping subscale in the current study sample was  .88.

#### 2.3.6. Mini-international Neuropsychiatric Interview (MINI [57])

The MINI is a brief clinical interview designed to screen for specific DSM-IV Axis I disorders. The MINI was used to screen for schizophrenia, schizoaffective disorder, and bipolar disorder for exclusionary criteria in this study. The MINI has good interrater and test-retest reliability, and convergent validity with the Structured Clinical Interview for DSM-III-R Patients (SCID-P) [58].

### 2.4. Statistical Analyses

Prior to testing hypotheses, simple unconditional analyses were conducted for African American, Hispanic, and white participants to obtain the treatment rate differences for the outcomes of participation in all secular mental health treatment, treatment with medication, psychotherapy treatment (individual and group), and religious counseling. Treatment rate difference is provided to convey meaningful clinical information [59].

After obtaining the treatment rate differences, all hypotheses were tested using multiple logistic regression analyses with the exception of the third hypothesis, which was tested using multiple linear regression. In addition to racial/ethnic identity—the primary independent variable—age, gender, annual income, hours worked per week in the last three months, and years of education were included as covariates to statistically account for sociodemographic factors.

## 3. Results

One-way ANOVAs testing group differences for covariates revealed significant group differences for annual income level, *F*(2,145) = 5.04, *P* = .008. Post hoc comparisons using the Tukey HSD test indicated that annual income was higher for white than for Hispanic participants at the .05 level of significance. All other comparisons were not significant. To determine if there were any differences between Hispanic participants who also identified as white (*n* = 19) and Hispanics who did not identify as white (*n* = 21), we conducted a series of *t*-tests. Comparisons indicated no significant differences on any predictor or outcome variables.

Of those who provided information on mental health treatment during the past four months, 50.7 percent reported using any specialty mental health services (individual or group psychotherapy or treatment with psychotropic medication) during the past four months. During that time period, 41.2 percent reported attending individual or group psychotherapy, 42.2 percent reported attending medication treatment, and 14.3 percent reported attending religious counseling. To characterize the sample, the treatment rate difference was obtained to compare the rates of treatment attendance between the racial/ethnic groups, whites versus African Americans and Hispanics, without controlling for symptom severity or sociodemographic characteristics (Table 2). Nonparametric tests of unconditional racial/ethnicity group differences were not significant for the use of any specialty mental health services [*χ* ^2^(2, *N* = 148) = 1.08, *P* = .58], individual/group psychotherapy attendance, [*χ* ^2^(2, *N* = 148) = 0.92, *P* = .63], medication treatment attendance, [*χ* ^2^(2, *N* = 147) = 1.36, *P* = .51], or use of religious counseling, [*χ* ^2^(2, *N* = 147), 0.73, *P* = .69]. 

A multiple logistic regression analysis was conducted to assess whether whites were more likely than either Blacks or Hispanics to attend any specialty mental health treatment (individual/group psychotherapy or medication treatment) when controlling for depressive and PTSD symptom severity and sociodemographic factors. All predictors were entered simultaneously. The overall model was significant [*χ* ^2^(9, *N* = 148) = 74.66, *P* < .001], and the Hosmer-Lemeshow Goodness of Fit Test supported the model [*χ* ^2^(8, *N* = 148) = 5.08, *P* = .75]. The model as a whole correctly classified 79.1% of cases. As shown in Table 3, there were no significant differences in odds of attending specialty mental health treatment as a function of race/ethnicity. However, female gender and higher PTSD symptomatology were associated with significantly higher odds of treatment attendance. 

A follow-up analysis was conducted to determine if there were racial/ethnic group differences in participation in individual/group psychotherapy. The overall model was significant [*χ* ^2^(9, *N* = 148) = 63.44, *P* < .001], and the Hosmer-Lemeshow Goodness of Fit Test supported the model [*χ* ^2^(8, *N* = 148) = 3.53, *P* = .90]. The model as a whole correctly classified 78.4% of cases. As shown in Table 4, higher depressive (OR = 1.07, 95% CI = 1.02–1.13) and PTSD (OR = 1.05, 95% CI = 1.01–1.08) symptoms were associated with significantly higher odds of attending individual/group psychotherapy.

A second follow-up analysis was conducted to examine potential differences in participation in mental health medication treatment. The overall model was significant [*χ* ^2^(9, *N* = 147) = 70.13, *P* < .001], and the Hosmer-Lemeshow Goodness of Fit Test supported the model [*χ* ^2^(8, *N* = 148) = 6.40, *P* = .60]. The model as a whole correctly classified 81.0% of cases. As shown in Table 5, higher PTSD symptoms and female gender were associated with significantly higher odds of attending medication treatment.

For the second hypothesis, a multiple logistic regression was performed to assess whether African Americans or Hispanics were more likely than whites to seek religious counseling from a priest, minister, or other religious figure when controlling for depression and posttraumatic stress symptom severity and sociodemographic variables. The full model containing all predictors was not statistically significant [*χ* ^2^(9, *N* = 147) = 7.20, *P* = .62].

To test the third hypothesis, a multiple linear regression analysis was performed to examine race/ethnicity as a predictor of religious coping. All predictors were entered simultaneously. The full model was statistically significant [*R* ^2^ = .179, *F*(9,138) = 3.33, *P* < .001]. As hypothesized, African American racial status predicted higher levels of religious coping compared to white racial status (See Table 6). Greater age also was associated with higher levels of religious coping.

For the fourth hypothesis, a multiple logistic regression was conducted to assess whether religious coping was associated with increased likelihood of using religious counseling. The overall model was significant [*χ* ^2^(10, *N* = 147) = 20.48, *P* < .05] and was able to correctly classify 86.4% of cases. A nonsignificant value was obtained from the Hosmer-Lemeshow Goodness of Fit test, providing support for the model's fit [*χ* ^2^(8, *N* = 147) = 9.74, *P* = .28]. As seen in Table 7, higher levels of religious coping were associated with increased odds of seeking religious counseling.

For the fifth hypothesis, a logistic regression analysis was conducted to test whether higher levels of religious coping were associated with lower odds of participation in specialty mental health treatment. The full model produced a good fit [*χ* ^2^(10, *N* = 148) = 77.01, *P* < .001], was supported by a nonsignificant Hosmer-Lemeshow Goodness of Fit Test [*χ* ^2^(8, *N* = 148) = 8.11, *P* = .42], and correctly classified 80.4% of cases. As seen in Table 8, higher PTSD symptom severity and female gender were significantly associated with increased odds of attending specialty mental health care. However, religious coping was not a statistically significant predictor (*P* = .13), although the zero-order correlation between use of specialty mental health treatment and level of religious coping indicated a significant but weak inverse relationship [*r* = −.20, *P* = .018]. Participation in secular mental health treatment in religious counseling were not significantly related [*r* = .06, *P* = .51].

## 4. Discussion

This study compared the use of mental health treatment among African Americans, Hispanic, and white OEF/OIF Veterans and evaluated whether differences in religious coping affected treatment involvement. Contrary to expectations, no significant differences were found as a function of race/ethnicity on the use of specialty mental health treatment—individual and/or group psychotherapy or medication treatment. Although these results are at odds with studies in the general population that found differences between whites and African Americans in use of mental health services [4, 5], they are consistent with results from several studies conducted within the VA that did not find differences in use of mental health services by whites and African Americans [10, 14, 15, 17]. Concerning the use of services by Hispanic Veterans, the current study's results are consistent with Rosenheck and Fontana [10], who found no differences between Hispanics and whites in the use of VA mental health services. Results lend support to the interpretation of Elhai et al. [14] that need for services appears to be a far stronger predictor than race or ethnicity of use of mental health services in the VA. Further, as suggested by Elhai et al. [14], young Veterans with disadvantaged socioeconomic status may not face the same problems as non-Veterans accessing mental health care because of the availability of VA services.

Consistent with studies of general community and college student samples, we found that women were more likely to seek specialty mental health treatment than men [60–62]. However, findings conflict with other VA studies that indicate that women Veterans are less likely than men to seek treatment within the VA [63, 64] or they are no more or less likely to use mental health services compared to men [65]. One explanation for discrepant results is that younger women Veterans who have served in OEF/OIF may be more comfortable using VA mental health services. Possible reasons for increased comfort include the growing percentage of women serving in the military and the VA's increased efforts to welcome women Veterans (e.g., by creating women's primary care clinics).

Interestingly, when psychotherapy and medication treatment were considered separately, female gender was positively associated with seeking medication treatment but not with psychotherapy attendance. However, the small number of women in our sample may have limited power to detect differences in follow-up analyses. After controlling for PTSD symptom level, depressive symptoms were a significant predictor in only one analysis: participation in psychotherapy. Thus, depression may be especially likely to lead people to seek out emotional support from others.

Contrary to the study's hypothesis, but consistent with Rosenheck and Fontana [10], data from the current study indicated that African Americans and Hispanics in the sample were not more likely than whites to seek religious counseling from a priest, minister, or religious figure. However, as predicted, African Americans reported higher levels of religious coping than whites. Results are consistent with previous research [33–37] and indicate that African American OEF/OIF Veterans reported greater use of prayer or meditation and finding comfort in religion when confronted with stressful events. Contrary to expectations and previous findings [36, 38, 39], Hispanics did not report higher levels of religious coping than whites. Consistent with past research indicating increased religious involvement with age [66–68], we found that older age was associated with higher religious coping. In contrast to findings indicating greater religious coping and religiosity among women [35, 69], we did not find female gender to be a significant predictor.

As expected, the use of religious coping was positively associated with seeking spiritual counseling, but contrary to expectations, was unrelated to seeking specialty mental health services (i.e., treatment with medication or individual/group psychotherapy). Veterans who said that they frequently pray or meditate or find comfort in their religion in response to stressful events were more likely to seek counseling from a religious figure. This finding is consistent with research that indicates the frequency of church attendance is positively related to a preference for religious counseling [22, 23]. Although African American racial status was associated with higher levels of religious coping, it was not significantly associated with greater likelihood of seeking religious counseling. The finding that use of religious coping did not predict use of specialty mental health services may indicate that reliance on private religious practices does not reduce seeking psychotherapy or mental health medication treatment. Further, participation in religious counseling over the previous four months was unrelated to use of specialty mental health services in our sample, *r* = .06, *P* > .05.

## 5. Limitations and Strengths

One important limitation is that the study did not employ random sampling and, therefore, inferences cannot be drawn about the larger population of OEF/OIF Veterans. Generalizability was also limited somewhat by the study's exclusion criteria. To increase the generalizability of findings, future treatment utilization studies would benefit from inclusion of Veterans with psychotic disorders or bipolar disorder, those who are at imminent suicide risk, and those who are not yet stable on medication or in psychotherapy. 

A further limitation is the use of secondary data analyses with a relatively small sample. Although we were able to detect racial/ethnicity differences in use of religious coping and gender differences in use of secular mental health services, future studies that include larger samples of OEF/OIF Veterans would allow for more powerful tests of possible racial/ethnic differences in treatment-seeking behavior. It should be noted that the SERVE project over-recruited Veterans with a history of psychiatric diagnoses, which enabled us to examine use of mental health services in a sample that had a high need for those services. We believe that these exploratory analyses are an important initial step toward determining how religious coping and race/ethnicity may impact the use of mental health services among OEF/OIF Veterans reporting relatively high levels of depression and PTSD symptoms.

Treatment involvement was reported via self report for the past four months rather than for lifetime use of services. This shorter time frame may have increased the measure's reliability, and a fairly large percentage of the sample reported using specialty mental health services (51%). However, the use of a longer retrospective time period would likely have resulted in a larger percentage of the sample reporting treatment use. Moreover, future studies should compare findings based on self-report versus actual chart review of service use. Prospective studies of service utilization are indicated.

Finally, the religious coping subscale from the Brief COPE contains only two items associated with positive religious coping and, therefore, did not measure the full spectrum of religious coping, including attendance of religious services/functions. The inclusion of a more comprehensive measure in future studies would enable a more detailed examination of negative and positive aspects of religious coping.

## 6. Research and Clinical Implications

The failure to find race/ethnicity differences in the use of mental health services in a sample of relatively young Veterans is a positive sign and suggests that individuals who might otherwise have limited access to mental health services in the community are taking advantage of those services in the VA. To ensure that minority groups access mental health services at similar levels as whites, it may be important to understand the relative importance of factors contributing to minority members' decision to seek care in the VA. Similarly, the current study's finding of greater use of mental health services among female than male Veterans is encouraging given past findings of lower use by women. If women are, in fact, seeking out more VA mental health services than in the past, it would be valuable to understand what factors are associated with this change. Future studies should also examine whether symptom severity moderates the association between race/ethnicity and mental health service use. 

The finding of higher religious coping among African American Veterans underlines the importance of assessing for religious coping and incorporating it into treatment plans, particularly when treating African Americans. It will be important for future research to determine whether treatment that incorporates religious practices and beliefs may be more acceptable to Veterans who report high levels of religious coping than treatment that focuses only on nonreligious behaviors and coping strategies. It may also be of value to examine the role religious coping plays by itself or in conjunction with evidence-based treatment in recovery from depression and traumatic events. 

##  Disclosure

The views expressed in this paper are those of the authors and do not necessarily reflect the position or policy of the Department of Veterans Affairs or the United States government.

## Figures and Tables

**Table 1:** Final sample characteristics.

	Final sample (*N* = 148)		African Americans (*N* = 30)		Hispanics (*N* = 40)		Whites (*N* = 78)	
Variable	M	SD	M	SD	M	SD	M	SD
Age	38.89	10.10	42.47	8.67	37.41	10.49	38.25	10.20
Years of education	14.28	2.51	14.00	2.36	14.15	2.84	14.46	2.40
Hours worked/week past 3 months	25.48	24.39	26.06	26.85	18.75	25.13	28.58	22.64
BDI-II	17.10	12.98	17.93	10.87	18.67	12.70	16.01	13.87
PCL-M	45.49	19.66	49.63	16.26	48.64	18.47	42.48	21.02

BDI-II: Beck Depression Inventory II; PCL-M: Posttraumatic Stress Disorder Checklist-Military Version.

**Table 2:** Mental health treatment rate differences by racial/ethnic group.

	Attended?		Total	Treatment rate	Treatment rate Difference (compared to white)
	Yes	No			
Any specialty mental health					

White	37	41	78	47.4%	
African American	15	15	30	50.0%	+2.6%
Hispanic	23	17	40	57.5%	+10.1%

Psychotherapy (individual/group)					

White	30	48	78	38.5%	
African American	12	18	30	40.0%	+1.5%
Hispanic	19	21	40	47.5%	+9.0%

Medication treatment					

White	29	48	77	37.7%	
African American	14	16	30	46.7%	+9.0%
Hispanic	19	21	40	47.5%	+9.8%

Religious counseling					

White	12	66	78	15.4%	
African American	5	25	30	16.7%	+1.3%
Hispanic	4	35	39	10.3%	−5.1%

**Table 3:** Logistic regression predicting any specialty mental health treatment attendance.

Predictor variable	*P*	Odds ratio (95% CI)
African Americans (*N* = 30)	.22	0.45 (0.13–1.62)
Hispanics (*N* = 40)	.90	1.07 (0.37–3.06)
Depression (BDI-II)	.12	1.05 (0.99–1.11)
PTSD (PCL-M)	.001***	1.07 (1.03–1.11)
Female (*N* = 23)	.009**	7.02 (1.63–30.35)
Age	.69	0.99 (0.94–1.04)
Years of education	.13	0.81 (0.63–1.06)
Hours worked per week	.79	1.00 (0.98–1.02)
Annual income	.33	1.22 (0.82–1.80)

CI: Confidence Interval.

*P < .05, **P < .01, ***P < .001.

**Table 4:** Logistic regression predicting individual and group psychotherapy.

Predictor variable	*P*	Odds ratio (95% CI)
African Americans (*N* = 30)	.50	0.66 (0.20–2.20)
Hispanics (*N* = 40)	.79	1.15 (0.42–3.11)
Depression (BDI-II)	.01*	1.07 (1.02–1.13)
PTSD (PCL-M)	.005**	1.05 (1.01–1.08)
Female (*N* = 23)	.61	1.41 (0.38–5.25)
Age	.83	1.00 (0.95–1.04)
Years of education	.68	0.95 (0.75–1.20)
Hours worked per week	.54	1.00 (0.98–1.01)
Annual income	.51	1.13 (0.80–1.61)

CI: Confidence Interval.

*P < .05, **P < .01, ***P < .001.

**Table 5:** Logistic regression predicting medication treatment.

Predictor variable	*P*	Odds ratio (95% CI)
African Americans (*N* = 30)	.62	0.73 (0.21–2.52)
Hispanics (*N* = 40)	.86	1.10 (0.40–3.07)
Depression (BDI-II)	.62	1.01 (0.96–1.07)
PTSD (PCL-M)	.001***	1.08 (1.04–1.12)
Female (*N* = 23)	.004**	8.77 (2.00–38.60)
Age	.67	0.99 (0.94–1.04)
Years of education	.12	0.81 (0.61–1.06)
Hours worked per week	.71	1.00 (0.98–1.02)
Annual income	.25	1.26 (0.85–1.87)

CI: Confidence Interval.

*P < .05, **P < .01, ***P < .001.

**Table 6:** Regression analysis summary for minority status as a predictor of religious coping.

Predictor variable	B	SE	*β*
African Americans	.63	.25	.23*
Hispanics	.24	.21	.10
Depression (BDI-II)	−.01	.01	−.07
PTSD (PCL-M)	−.01	.01	−.09
Female	.37	.26	.12
Age	.02	.01	.21*
Years of education	.06	.04	.13
Hours worked per week	.00	.00	−.01
Annual income	−.09	.08	−.12

*R* ^2^ = .179 (*N* = 148, *P* < .001).

*P < .05, **P < .01, ***P < .001.

**Table 7:** Logistic regression with religious coping as a predictor of religious counseling attendance.

Predictor variable	*P*	Odds ratio (95% CI)
Religious coping	.001***	2.75 (1.49–5.07)
African Americans (*N* = 30)	.35	0.50 (0.12–2.10)
Hispanics (*N* = 39)	.21	0.41 (0.10–1.63)
Depression (BDI-II)	.21	1.04 (0.98–1.10)
PTSD (PCL-M)	.70	1.01 (0.97–1.05)
Female (*N* = 22)	.43	1.73 (0.44–6.82)
Age	.24	0.96 (0.90–1.03)
Years of education	.40	0.89 (0.67–1.17)
Hours worked per week	.89	1.00 (0.98–1.03)
Annual income	.77	1.07 (0.69–1.67)

*P < .05, **P < .01, ***P < .001.

**Table 8:** Logistic regression with religious coping as a predictor of use of specialty mental health treatment.

Predictor variable	*P*	Odds ratio (95% CI)
Religious coping	.13	0.71 (0.46–1.10)
African Americans (*N* = 30)	.37	0.55 (0.15–2.06)
Hispanics (*N* = 40)	.77	1.17 (0.40–3.44)
Depression (BDI-II)	.12	1.05 (0.99–1.11)
PTSD (PCL-M)	.001***	1.07 (1.03–1.11)
Female (*N* = 23)	.006**	8.06 (1.82–35.70)
Age	.96	1.00 (0.95–1.05)
Years of education	.21	0.84 (0.65–1.10)
Hours worked per week	.84	1.00 (0.98–1.02)
Annual income	.35	1.21 (0.81–1.80)

*P < .05, **P < .01, ***P < .001.
